# *trans*-Tetra­kis[*N*-(adamantan-1-yl)pyridine-4-carboxamide]­dichlorido­manganese(II)–*N*-(adamantan-1-yl)pyridine-4-carboxamide (1/2)

**DOI:** 10.1107/S1600536812010124

**Published:** 2012-03-14

**Authors:** Ying-Chun Wang

**Affiliations:** a College of Chemistry and Chemical Engineering, Southeast University, Nanjing 210096, People’s Republic of China

## Abstract

The asymmetric unit of the title compound, [MnCl_2_(C_16_H_20_N_2_O)_4_]·2C_16_H_20_N_2_O, is composed of two coordinating *N*-(adamantan-1-yl)pyridine-4-carboxamide mol­ecules, one Cl^−^ anion, an Mn^II^ ion, lying on an inversion centre, and one free *N*-(adamantan-1-yl)pyridine-4-carboxamide mol­ecule. The distorted octa­hedral Mn environment comprises two terminal Cl atoms and four monodentate N atoms from four organic ligands. All the carbamoyl N atoms are involved in inter­molecular N—H⋯O hydrogen-bonding inter­actions which link the mol­ecules into a chain along the *a* axis.

## Related literature

For the structures of related amino compounds, see: Fu *et al.* (2007 ▶, 2008 ▶, 2009 ▶); Fu & Xiong (2008 ▶). For the ferroelectric properties of related amino derivatives, see: Fu *et al.* (2011*a* ▶,*b* ▶,*c* ▶).
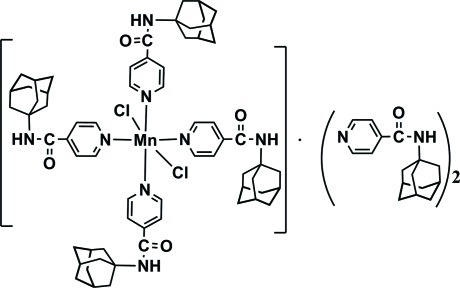


## Experimental

### 

#### Crystal data

[MnCl_2_(C_16_H_20_N_2_O)_4_]·2C_16_H_20_N_2_O
*M* *_r_* = 1663.88
Triclinic, 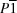

*a* = 11.189 (4) Å
*b* = 11.571 (4) Å
*c* = 16.921 (6) Å
α = 86.122 (9)°
β = 83.605 (9)°
γ = 84.094 (10)°
*V* = 2162.0 (13) Å^3^
*Z* = 1
Mo *K*α radiation
μ = 0.28 mm^−1^
*T* = 298 K
0.30 × 0.25 × 0.15 mm

#### Data collection

Rigaku Mercury2 diffractometer
Absorption correction: multi-scan (*CrystalClear*; Rigaku, 2005 ▶) *T*_min_ = 0.90, *T*_max_ = 1.00
18634 measured reflections
9700 independent reflections
6263 reflections with *I* > 2σ(*I*)
*R*_int_ = 0.056

#### Refinement

*R*[*F* ^2^ > 2σ(*F* ^2^)] = 0.078
*wR*(*F* ^2^) = 0.164
*S* = 1.05
9700 reflections
529 parameters
H-atom parameters constrained
Δρ_max_ = 0.28 e Å^−3^
Δρ_min_ = −0.38 e Å^−3^

### 

Data collection: *CrystalClear* (Rigaku, 2005 ▶); cell refinement: *CrystalClear*; data reduction: *CrystalClear*; program(s) used to solve structure: *SHELXTL* (Sheldrick, 2008 ▶); program(s) used to refine structure: *SHELXTL*; molecular graphics: *SHELXTL*; software used to prepare material for publication: *SHELXTL*.

## Supplementary Material

Crystal structure: contains datablock(s) I, global. DOI: 10.1107/S1600536812010124/gw2114sup1.cif

Structure factors: contains datablock(s) I. DOI: 10.1107/S1600536812010124/gw2114Isup2.hkl

Additional supplementary materials:  crystallographic information; 3D view; checkCIF report

## Figures and Tables

**Table 1:** Hydrogen-bond geometry (Å, °)

*D*—H⋯*A*	*D*—H	H⋯*A*	*D*⋯*A*	*D*—H⋯*A*
N4—H4*B*⋯O1^i^	0.86	2.18	3.024 (4)	168
N2—H2*B*⋯O3^ii^	0.86	2.25	3.064 (4)	157

Symmetry codes: (i) 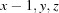; (ii) 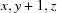.

**Fig. 1.**
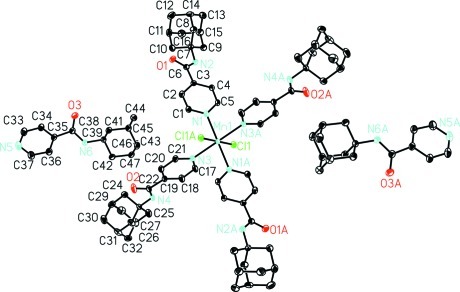
Molecular view of the title compound with the atomic numbering scheme. Displacement ellipsoids are drawn at the 30% probability level. H atoms have been omitted for clarity.

**Fig. 2.**
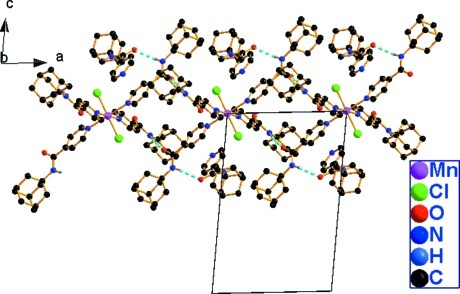
The crystal packing of the title compound viewed along the b axis showing the hydrogen bonding chain (dashed line). H atoms not involved in hydrogen bonding (dashed line) have been omitted for clarity.

## supplementary crystallographic information

### Comment

Amino compounds attracted more attention as phase transition dielectric materials for its application in memory storage (Fu *et al.* 2007; Fu & Xiong 2008; Fu *et al.* 2008; Fu *et al.* 2009). With the purpose of obtaining phase transition crystals of amino compounds, various amines have been studied and we have elaborated a series of new materials with this organic molecules (Fu *et al.*, 2011*a*, *b*, *c*). In this study, we describe the crystal structure of the title compound, *trans*-tetrakis{*N*-(adamantan-1-yl)pyridine-4-carboxamide}dichloridomanganese(II)–*N*-(adamantan-1-yl)pyridine-4-carboxamide (1/2).

The asymmetric unit is composed of two coordinated *N*-(adamantan-1-yl)pyridine-4-carboxamide molecules, one Cl^-^ anion, half Mn^II^ ion and one free *N*-(adamantan-1-yl)pyridine-4-carboxamide molecule. The Mn atom lies on an inversion center. The distorted octahedral Mn environment contains two terminal Cl atoms and four monodentate N atoms from four organic ligand. The geometric parameters of the title compound are in the normal range.

In the crystal structure, all the carbamoyl N atoms (N2 and N4) are involved in intermolecular N—H···O H-bonding interactions with the O1 and O3 atoms, respectively. These hydrogen bonds link the molecular units into a one-dimensional chain along the *a*-axis(Table 1 and Fig.2).

### Experimental

MnCl_2_.6H_2_O (2 mmol) and *N*-(adamantan-1-yl)pyridine-4-carboxamide (2 mmol) were dissolved in 70% methanol aqueous solution, and then 2 ml HCl was added. Single crystals suitable for X-ray diffraction analysis were obtained from slow evaporation of the solution at room temperature after two weeks.

### Refinement

All H atoms attached to C and N atoms were fixed geometrically and treated as riding with C-H = 0.93 Å (aromatic), C-H = 0.97 Å (methylene), C-H = 0.98 Å (methine) and N-H = 0.86 Å (N), with *U*_iso_(H) = 1.2*U*_eq_(C and N).

### Crystal data

**Table:**

[MnCl_2_(C_16_H_20_N_2_O)_4_]·2C_16_H_20_N_2_O	*Z* = 1
*M**_r_* = 1663.88	*F*(000) = 887
Triclinic, *P*1	*D*_x_ = 1.278 Mg m^−^^3^
Hall symbol: -P 1	Mo *K*α radiation, λ = 0.71073 Å
*a* = 11.189 (4) Å	Cell parameters from 9701 reflections
*b* = 11.571 (4) Å	θ = 3.2–27.5°
*c* = 16.921 (6) Å	µ = 0.28 mm^−^^1^
α = 86.122 (9)°	*T* = 298 K
β = 83.605 (9)°	Block, colorless
γ = 84.094 (10)°	0.30 × 0.25 × 0.15 mm
*V* = 2162.0 (13) Å^3^	

### Data collection

**Table:**

Rigaku Mercury2 diffractometer	9700 independent reflections
Radiation source: fine-focus sealed tube	6263 reflections with *I* > 2σ(*I*)
Graphite monochromator	*R*_int_ = 0.056
Detector resolution: 13.6612 pixels mm^-1^	θ_max_ = 27.5°, θ_min_ = 3.2°
CCD profile fitting scans	*h* = −13→14
Absorption correction: multi-scan (*CrystalClear*; Rigaku, 2005)	*k* = −14→14
*T*_min_ = 0.90, *T*_max_ = 1.00	*l* = −21→21
18634 measured reflections	

### Refinement

**Table:**

Refinement on *F*^2^	Primary atom site location: structure-invariant direct methods
Least-squares matrix: full	Secondary atom site location: difference Fourier map
*R*[*F*^2^ > 2σ(*F*^2^)] = 0.078	Hydrogen site location: inferred from neighbouring sites
*wR*(*F*^2^) = 0.164	H-atom parameters constrained
*S* = 1.05	*w* = 1/[σ^2^(*F*_o_^2^) + (0.0517*P*)^2^ + 0.6*P*] where *P* = (*F*_o_^2^ + 2*F*_c_^2^)/3
9700 reflections	(Δ/σ)_max_ = 0.001
529 parameters	Δρ_max_ = 0.28 e Å^−^^3^
0 restraints	Δρ_min_ = −0.38 e Å^−^^3^

### Special details

**Table:**

Geometry. All esds (except the esd in the dihedral angle between two l.s. planes) are estimated using the full covariance matrix. The cell esds are taken into account individually in the estimation of esds in distances, angles and torsion angles; correlations between esds in cell parameters are only used when they are defined by crystal symmetry. An approximate (isotropic) treatment of cell esds is used for estimating esds involving l.s. planes.
Refinement. Refinement of F^2^ against ALL reflections. The weighted R-factor wR and goodness of fit S are based on F^2^, conventional R-factors R are based on F, with F set to zero for negative F^2^. The threshold expression of F^2^ > 2sigma(F^2^) is used only for calculating R-factors(gt) etc. and is not relevant to the choice of reflections for refinement. R-factors based on F^2^ are statistically about twice as large as those based on F, and R- factors based on ALL data will be even larger.

### Fractional atomic coordinates and isotropic or equivalent isotropic displacement parameters (Å^2^)

**Table:**

	*x*	*y*	*z*	*U*_iso_*/*U*_eq_	
Mn1	0.0000	1.0000	0.0000	0.02697 (18)	
Cl1	−0.10718 (7)	1.06669 (7)	0.12815 (5)	0.0346 (2)	
O2	−0.2281 (2)	0.4241 (2)	0.01989 (17)	0.0571 (8)	
O1	0.5058 (2)	0.7112 (2)	0.19793 (14)	0.0396 (6)	
N1	0.1600 (2)	0.9277 (2)	0.06876 (15)	0.0308 (6)	
N3	−0.0941 (2)	0.8243 (2)	0.01766 (16)	0.0323 (6)	
N4	−0.3668 (2)	0.5025 (2)	0.11487 (16)	0.0353 (7)	
H4B	−0.3924	0.5654	0.1383	0.042*	
N2	0.4189 (2)	0.8268 (2)	0.29637 (15)	0.0348 (7)	
H2B	0.3592	0.8771	0.3096	0.042*	
C12	0.7255 (4)	0.6679 (4)	0.4100 (3)	0.0627 (12)	
H12A	0.7814	0.6470	0.4497	0.075*	
H12B	0.7561	0.6280	0.3622	0.075*	
C27	−0.5991 (3)	0.3238 (3)	0.2344 (2)	0.0491 (10)	
H27A	−0.6614	0.3444	0.2779	0.059*	
C26	−0.4925 (3)	0.3603 (3)	0.0703 (2)	0.0463 (10)	
H26A	−0.4324	0.3419	0.0260	0.056*	
H26B	−0.5491	0.4234	0.0526	0.056*	
C28	−0.5115 (4)	0.2239 (4)	0.2630 (2)	0.0566 (11)	
H28A	−0.4739	0.2472	0.3076	0.068*	
H28B	−0.5552	0.1571	0.2808	0.068*	
C6	0.4266 (3)	0.7862 (3)	0.22306 (19)	0.0300 (7)	
C3	0.3321 (3)	0.8378 (3)	0.17077 (18)	0.0277 (7)	
C29	−0.4154 (3)	0.1916 (3)	0.1958 (2)	0.0504 (10)	
H29A	−0.3593	0.1274	0.2143	0.060*	
C15	0.5432 (3)	0.8246 (3)	0.4959 (2)	0.0488 (10)	
H15A	0.5117	0.8652	0.5439	0.059*	
C19	−0.2141 (3)	0.6209 (3)	0.04641 (19)	0.0301 (7)	
C23	−0.4314 (3)	0.3980 (3)	0.13873 (18)	0.0280 (7)	
C10	0.5145 (3)	0.6604 (3)	0.3768 (2)	0.0437 (9)	
H10A	0.4353	0.6365	0.3961	0.052*	
H10B	0.5444	0.6188	0.3296	0.052*	
C7	0.5046 (3)	0.7918 (3)	0.35582 (19)	0.0302 (7)	
C5	0.1945 (3)	0.9920 (3)	0.12394 (18)	0.0296 (7)	
H5A	0.1597	1.0682	0.1278	0.036*	
C4	0.2785 (3)	0.9511 (3)	0.17497 (18)	0.0293 (7)	
H4A	0.2993	0.9991	0.2122	0.035*	
C1	0.2135 (3)	0.8189 (3)	0.06393 (19)	0.0337 (8)	
H1A	0.1921	0.7732	0.0256	0.040*	
C21	−0.0561 (3)	0.7270 (3)	−0.0209 (2)	0.0337 (8)	
H21A	0.0125	0.7279	−0.0573	0.040*	
C13	0.6669 (4)	0.8629 (4)	0.4657 (2)	0.0551 (11)	
H13A	0.6605	0.9462	0.4534	0.066*	
H13B	0.7215	0.8452	0.5065	0.066*	
C32	−0.6582 (3)	0.2870 (3)	0.1641 (3)	0.0525 (11)	
H32A	−0.7045	0.2216	0.1808	0.063*	
H32B	−0.7128	0.3508	0.1450	0.063*	
C11	0.5522 (4)	0.6940 (4)	0.5157 (2)	0.0562 (11)	
H11A	0.6057	0.6742	0.5569	0.067*	
H11B	0.4731	0.6704	0.5354	0.067*	
C14	0.7157 (3)	0.7992 (4)	0.3915 (2)	0.0511 (10)	
H14A	0.7959	0.8230	0.3723	0.061*	
C2	0.2988 (3)	0.7711 (3)	0.11291 (19)	0.0343 (8)	
H2A	0.3335	0.6952	0.1073	0.041*	
C18	−0.2537 (3)	0.7206 (3)	0.0852 (2)	0.0434 (9)	
H18A	−0.3221	0.7220	0.1220	0.052*	
C20	−0.1131 (3)	0.6260 (3)	−0.0092 (2)	0.0348 (8)	
H20A	−0.0842	0.5618	−0.0384	0.042*	
C24	−0.3459 (3)	0.2982 (3)	0.1693 (2)	0.0436 (9)	
H24A	−0.3091	0.3224	0.2140	0.052*	
H24B	−0.2821	0.2777	0.1276	0.052*	
C22	−0.2715 (3)	0.5064 (3)	0.0597 (2)	0.0352 (8)	
C9	0.4562 (3)	0.8559 (3)	0.43119 (19)	0.0395 (8)	
H9A	0.3765	0.8335	0.4506	0.047*	
H9B	0.4494	0.9392	0.4188	0.047*	
C8	0.6304 (3)	0.8299 (3)	0.3259 (2)	0.0436 (9)	
H8A	0.6244	0.9131	0.3129	0.052*	
H8B	0.6624	0.7905	0.2782	0.052*	
C30	−0.4731 (4)	0.1551 (3)	0.1260 (3)	0.0563 (11)	
H30A	−0.4111	0.1350	0.0830	0.068*	
H30B	−0.5156	0.0870	0.1419	0.068*	
C25	−0.5301 (3)	0.4300 (3)	0.2067 (2)	0.0501 (10)	
H25A	−0.4934	0.4557	0.2510	0.060*	
H25B	−0.5858	0.4934	0.1884	0.060*	
C31	−0.5601 (4)	0.2528 (4)	0.0976 (2)	0.0517 (11)	
H31A	−0.5977	0.2281	0.0527	0.062*	
C16	0.6009 (4)	0.6307 (3)	0.4413 (2)	0.0534 (11)	
H16A	0.6074	0.5465	0.4541	0.064*	
C17	−0.1922 (3)	0.8187 (3)	0.0698 (2)	0.0416 (9)	
H17A	−0.2209	0.8846	0.0973	0.050*	
O3	0.1737 (2)	−0.0581 (2)	0.36458 (15)	0.0453 (6)	
C35	0.0769 (3)	−0.1917 (3)	0.2992 (2)	0.0357 (8)	
N6	−0.0146 (2)	0.0022 (2)	0.32917 (18)	0.0391 (7)	
H6A	−0.0765	−0.0220	0.3119	0.047*	
C34	0.1500 (3)	−0.2864 (3)	0.3272 (2)	0.0423 (9)	
H34A	0.1949	−0.2792	0.3694	0.051*	
C39	−0.0263 (3)	0.1255 (3)	0.3506 (2)	0.0329 (8)	
C37	0.0227 (4)	−0.3182 (3)	0.2058 (2)	0.0549 (11)	
H37A	−0.0219	−0.3285	0.1640	0.066*	
C38	0.0828 (3)	−0.0759 (3)	0.3340 (2)	0.0358 (8)	
C44	0.0683 (3)	0.3344 (3)	0.3985 (2)	0.0447 (9)	
H44A	0.1511	0.3043	0.4047	0.054*	
H44B	0.0583	0.4158	0.4109	0.054*	
C36	0.0108 (3)	−0.2088 (3)	0.2371 (2)	0.0437 (9)	
H36A	−0.0405	−0.1484	0.2167	0.052*	
N5	0.0936 (3)	−0.4090 (3)	0.2317 (2)	0.0607 (10)	
C45	−0.0186 (3)	0.2659 (3)	0.4557 (2)	0.0421 (9)	
H45A	−0.0018	0.2736	0.5106	0.051*	
C41	0.0602 (3)	0.1944 (3)	0.2937 (2)	0.0352 (8)	
H41A	0.1430	0.1639	0.2994	0.042*	
H41B	0.0449	0.1865	0.2391	0.042*	
C43	0.0417 (3)	0.3231 (3)	0.3128 (2)	0.0370 (8)	
H43A	0.0970	0.3671	0.2763	0.044*	
C47	−0.1747 (3)	0.3028 (3)	0.3602 (2)	0.0405 (9)	
H47A	−0.2582	0.3336	0.3537	0.049*	
C42	−0.1569 (3)	0.1744 (3)	0.3411 (2)	0.0381 (8)	
H42A	−0.1743	0.1665	0.2869	0.046*	
H42B	−0.2121	0.1308	0.3767	0.046*	
C46	−0.1493 (3)	0.3157 (3)	0.4459 (2)	0.0470 (10)	
H46A	−0.2050	0.2741	0.4826	0.056*	
H46B	−0.1603	0.3972	0.4578	0.056*	
C40	−0.0016 (3)	0.1368 (3)	0.4371 (2)	0.0389 (8)	
H40A	−0.0568	0.0936	0.4731	0.047*	
H40B	0.0803	0.1048	0.4444	0.047*	
C33	0.1558 (4)	−0.3917 (3)	0.2921 (3)	0.0539 (11)	
H33A	0.2057	−0.4538	0.3116	0.065*	
C48	−0.0884 (3)	0.3711 (3)	0.3031 (2)	0.0430 (9)	
H48A	−0.1052	0.3645	0.2487	0.052*	
H48B	−0.1000	0.4528	0.3146	0.052*	

### Atomic displacement parameters (Å^2^)

**Table:**

	*U*^11^	*U*^22^	*U*^33^	*U*^12^	*U*^13^	*U*^23^
Mn1	0.0290 (4)	0.0249 (4)	0.0275 (4)	−0.0036 (3)	−0.0037 (3)	−0.0026 (3)
Cl1	0.0371 (5)	0.0329 (4)	0.0332 (4)	−0.0029 (4)	0.0010 (3)	−0.0066 (3)
O2	0.0589 (18)	0.0316 (14)	0.0768 (19)	−0.0162 (13)	0.0315 (15)	−0.0211 (14)
O1	0.0322 (13)	0.0436 (15)	0.0407 (13)	0.0091 (11)	0.0009 (11)	−0.0141 (11)
N1	0.0353 (16)	0.0278 (15)	0.0296 (14)	−0.0033 (12)	−0.0020 (12)	−0.0051 (12)
N3	0.0309 (15)	0.0263 (15)	0.0397 (15)	−0.0066 (12)	−0.0008 (12)	−0.0015 (12)
N4	0.0373 (16)	0.0245 (15)	0.0440 (16)	−0.0087 (13)	0.0059 (13)	−0.0091 (12)
N2	0.0335 (16)	0.0362 (16)	0.0343 (15)	0.0081 (13)	−0.0080 (12)	−0.0091 (13)
C12	0.054 (3)	0.070 (3)	0.066 (3)	0.021 (2)	−0.027 (2)	−0.019 (2)
C27	0.045 (2)	0.042 (2)	0.056 (2)	−0.0140 (19)	0.0217 (19)	−0.0065 (19)
C26	0.056 (2)	0.047 (2)	0.039 (2)	−0.022 (2)	−0.0076 (18)	0.0015 (17)
C28	0.065 (3)	0.062 (3)	0.044 (2)	−0.019 (2)	−0.008 (2)	0.012 (2)
C6	0.0283 (17)	0.0305 (18)	0.0313 (17)	−0.0041 (15)	−0.0023 (14)	−0.0032 (14)
C3	0.0234 (16)	0.0311 (17)	0.0278 (15)	−0.0008 (14)	−0.0002 (13)	−0.0024 (13)
C29	0.037 (2)	0.042 (2)	0.069 (3)	−0.0013 (18)	−0.002 (2)	0.018 (2)
C15	0.055 (2)	0.056 (3)	0.038 (2)	0.001 (2)	−0.0147 (18)	−0.0121 (18)
C19	0.0303 (18)	0.0232 (17)	0.0370 (17)	−0.0044 (14)	−0.0016 (14)	−0.0043 (14)
C23	0.0294 (17)	0.0241 (16)	0.0307 (16)	−0.0053 (14)	−0.0013 (13)	−0.0017 (13)
C10	0.051 (2)	0.034 (2)	0.049 (2)	−0.0042 (18)	−0.0156 (18)	−0.0031 (17)
C7	0.0293 (17)	0.0270 (17)	0.0350 (17)	0.0023 (14)	−0.0086 (14)	−0.0048 (14)
C5	0.0337 (18)	0.0202 (16)	0.0350 (17)	−0.0016 (14)	−0.0042 (14)	−0.0035 (13)
C4	0.0308 (18)	0.0272 (17)	0.0306 (16)	−0.0022 (14)	−0.0040 (14)	−0.0064 (13)
C1	0.040 (2)	0.0310 (18)	0.0318 (17)	0.0006 (15)	−0.0080 (15)	−0.0102 (14)
C21	0.0320 (19)	0.0271 (18)	0.0397 (18)	−0.0016 (15)	0.0054 (15)	−0.0032 (15)
C13	0.056 (3)	0.053 (3)	0.062 (3)	−0.005 (2)	−0.028 (2)	−0.012 (2)
C32	0.030 (2)	0.049 (2)	0.079 (3)	−0.0117 (18)	−0.009 (2)	0.012 (2)
C11	0.069 (3)	0.058 (3)	0.046 (2)	−0.008 (2)	−0.027 (2)	0.005 (2)
C14	0.028 (2)	0.061 (3)	0.068 (3)	−0.0049 (19)	−0.0140 (19)	−0.011 (2)
C2	0.038 (2)	0.0281 (18)	0.0354 (18)	0.0022 (15)	0.0003 (15)	−0.0056 (14)
C18	0.038 (2)	0.035 (2)	0.056 (2)	−0.0116 (17)	0.0150 (18)	−0.0144 (17)
C20	0.0322 (18)	0.0266 (18)	0.0439 (19)	−0.0030 (15)	0.0048 (15)	−0.0046 (15)
C24	0.035 (2)	0.038 (2)	0.058 (2)	−0.0095 (17)	−0.0068 (18)	0.0058 (18)
C22	0.037 (2)	0.0245 (17)	0.0439 (19)	−0.0073 (15)	0.0026 (16)	−0.0069 (15)
C9	0.041 (2)	0.044 (2)	0.0347 (18)	0.0004 (17)	−0.0073 (16)	−0.0096 (16)
C8	0.034 (2)	0.047 (2)	0.050 (2)	−0.0055 (17)	−0.0031 (17)	−0.0022 (18)
C30	0.058 (3)	0.038 (2)	0.073 (3)	−0.019 (2)	0.015 (2)	−0.014 (2)
C25	0.054 (2)	0.041 (2)	0.054 (2)	−0.0116 (19)	0.013 (2)	−0.0127 (19)
C31	0.060 (3)	0.056 (3)	0.045 (2)	−0.030 (2)	−0.009 (2)	−0.0064 (19)
C16	0.070 (3)	0.034 (2)	0.059 (3)	0.005 (2)	−0.031 (2)	−0.0014 (19)
C17	0.044 (2)	0.0290 (19)	0.052 (2)	−0.0093 (17)	0.0081 (18)	−0.0119 (16)
O3	0.0409 (15)	0.0487 (16)	0.0465 (15)	0.0034 (12)	−0.0101 (12)	−0.0073 (12)
C35	0.0345 (19)	0.0294 (18)	0.0399 (19)	−0.0006 (15)	0.0048 (15)	0.0039 (15)
N6	0.0298 (16)	0.0314 (16)	0.0563 (19)	−0.0014 (13)	−0.0045 (14)	−0.0065 (14)
C34	0.046 (2)	0.033 (2)	0.045 (2)	−0.0001 (17)	0.0025 (17)	0.0042 (16)
C39	0.0320 (18)	0.0269 (17)	0.0404 (19)	−0.0037 (15)	−0.0049 (15)	−0.0035 (15)
C37	0.072 (3)	0.042 (2)	0.054 (2)	−0.007 (2)	−0.012 (2)	−0.004 (2)
C38	0.036 (2)	0.0339 (19)	0.0356 (18)	−0.0015 (16)	0.0004 (15)	0.0001 (15)
C44	0.045 (2)	0.042 (2)	0.049 (2)	−0.0068 (18)	−0.0095 (18)	−0.0053 (18)
C36	0.049 (2)	0.033 (2)	0.048 (2)	−0.0052 (17)	−0.0060 (18)	0.0041 (17)
N5	0.081 (3)	0.0349 (19)	0.066 (2)	−0.0094 (19)	−0.005 (2)	−0.0002 (17)
C45	0.050 (2)	0.045 (2)	0.0308 (18)	0.0020 (18)	−0.0056 (16)	−0.0054 (16)
C41	0.0315 (18)	0.0359 (19)	0.0362 (18)	−0.0010 (15)	0.0021 (15)	−0.0013 (15)
C43	0.038 (2)	0.0303 (19)	0.0411 (19)	−0.0059 (16)	0.0005 (16)	0.0039 (15)
C47	0.0321 (19)	0.039 (2)	0.048 (2)	0.0063 (16)	−0.0033 (16)	−0.0050 (17)
C42	0.0323 (19)	0.038 (2)	0.043 (2)	−0.0018 (16)	−0.0045 (16)	0.0010 (16)
C46	0.050 (2)	0.044 (2)	0.043 (2)	0.0007 (19)	0.0069 (18)	−0.0059 (18)
C40	0.041 (2)	0.036 (2)	0.0382 (19)	−0.0004 (17)	−0.0040 (16)	0.0036 (16)
C33	0.063 (3)	0.031 (2)	0.064 (3)	0.000 (2)	0.002 (2)	0.0073 (19)
C48	0.049 (2)	0.0302 (19)	0.048 (2)	0.0048 (17)	−0.0078 (18)	0.0030 (17)

### Geometric parameters (Å, º)

**Table:**

Mn1—N1	2.298 (3)	C32—H32B	0.9700
Mn1—N1^i^	2.298 (3)	C11—C16	1.520 (5)
Mn1—N3^i^	2.370 (3)	C11—H11A	0.9700
Mn1—N3	2.370 (3)	C11—H11B	0.9700
Mn1—Cl1	2.4885 (10)	C14—C8	1.542 (5)
Mn1—Cl1^i^	2.4885 (10)	C14—H14A	0.9800
O2—C22	1.230 (4)	C2—H2A	0.9300
O1—C6	1.236 (4)	C18—C17	1.383 (4)
N1—C1	1.341 (4)	C18—H18A	0.9300
N1—C5	1.345 (4)	C20—H20A	0.9300
N3—C17	1.333 (4)	C24—H24A	0.9700
N3—C21	1.346 (4)	C24—H24B	0.9700
N4—C22	1.339 (4)	C9—H9A	0.9700
N4—C23	1.482 (4)	C9—H9B	0.9700
N4—H4B	0.8600	C8—H8A	0.9700
N2—C6	1.347 (4)	C8—H8B	0.9700
N2—C7	1.475 (4)	C30—C31	1.504 (6)
N2—H2B	0.8600	C30—H30A	0.9700
C12—C14	1.526 (6)	C30—H30B	0.9700
C12—C16	1.529 (6)	C25—H25A	0.9700
C12—H12A	0.9700	C25—H25B	0.9700
C12—H12B	0.9700	C31—H31A	0.9800
C27—C28	1.525 (5)	C16—H16A	0.9800
C27—C32	1.528 (5)	C17—H17A	0.9300
C27—C25	1.537 (5)	O3—C38	1.232 (4)
C27—H27A	0.9800	C35—C34	1.388 (5)
C26—C23	1.518 (4)	C35—C36	1.387 (5)
C26—C31	1.539 (5)	C35—C38	1.509 (5)
C26—H26A	0.9700	N6—C38	1.348 (4)
C26—H26B	0.9700	N6—C39	1.486 (4)
C28—C29	1.515 (5)	N6—H6A	0.8600
C28—H28A	0.9700	C34—C33	1.383 (5)
C28—H28B	0.9700	C34—H34A	0.9300
C6—C3	1.504 (4)	C39—C41	1.532 (4)
C3—C4	1.387 (4)	C39—C42	1.533 (4)
C3—C2	1.391 (4)	C39—C40	1.537 (5)
C29—C30	1.511 (6)	C37—N5	1.329 (5)
C29—C24	1.540 (5)	C37—C36	1.394 (5)
C29—H29A	0.9800	C37—H37A	0.9300
C15—C13	1.520 (5)	C44—C45	1.534 (5)
C15—C11	1.521 (5)	C44—C43	1.530 (5)
C15—C9	1.545 (5)	C44—H44A	0.9700
C15—H15A	0.9800	C44—H44B	0.9700
C19—C18	1.375 (4)	C36—H36A	0.9300
C19—C20	1.391 (4)	N5—C33	1.335 (5)
C19—C22	1.524 (4)	C45—C40	1.536 (5)
C23—C24	1.523 (4)	C45—C46	1.538 (5)
C23—C25	1.540 (4)	C45—H45A	0.9800
C10—C16	1.536 (5)	C41—C43	1.533 (4)
C10—C7	1.533 (4)	C41—H41A	0.9700
C10—H10A	0.9700	C41—H41B	0.9700
C10—H10B	0.9700	C43—C48	1.526 (5)
C7—C9	1.536 (4)	C43—H43A	0.9800
C7—C8	1.540 (4)	C47—C48	1.528 (5)
C5—C4	1.374 (4)	C47—C42	1.530 (5)
C5—H5A	0.9300	C47—C46	1.529 (5)
C4—H4A	0.9300	C47—H47A	0.9800
C1—C2	1.382 (4)	C42—H42A	0.9700
C1—H1A	0.9300	C42—H42B	0.9700
C21—C20	1.381 (4)	C46—H46A	0.9700
C21—H21A	0.9300	C46—H46B	0.9700
C13—C14	1.520 (5)	C40—H40A	0.9700
C13—H13A	0.9700	C40—H40B	0.9700
C13—H13B	0.9700	C33—H33A	0.9300
C32—C31	1.524 (5)	C48—H48A	0.9700
C32—H32A	0.9700	C48—H48B	0.9700
			
N1—Mn1—N1^i^	180.000 (1)	C21—C20—H20A	120.2
N1—Mn1—N3^i^	86.06 (9)	C19—C20—H20A	120.2
N1^i^—Mn1—N3^i^	93.94 (9)	C23—C24—C29	110.2 (3)
N1—Mn1—N3	93.94 (9)	C23—C24—H24A	109.6
N1^i^—Mn1—N3	86.06 (9)	C29—C24—H24A	109.6
N3^i^—Mn1—N3	180.0	C23—C24—H24B	109.6
N1—Mn1—Cl1	88.08 (7)	C29—C24—H24B	109.6
N1^i^—Mn1—Cl1	91.92 (7)	H24A—C24—H24B	108.1
N3^i^—Mn1—Cl1	89.34 (7)	O2—C22—N4	124.3 (3)
N3—Mn1—Cl1	90.66 (7)	O2—C22—C19	118.7 (3)
N1—Mn1—Cl1^i^	91.92 (7)	N4—C22—C19	117.0 (3)
N1^i^—Mn1—Cl1^i^	88.08 (7)	C7—C9—C15	109.5 (3)
N3^i^—Mn1—Cl1^i^	90.66 (7)	C7—C9—H9A	109.8
N3—Mn1—Cl1^i^	89.34 (7)	C15—C9—H9A	109.8
Cl1—Mn1—Cl1^i^	180.0	C7—C9—H9B	109.8
C1—N1—C5	116.7 (3)	C15—C9—H9B	109.8
C1—N1—Mn1	123.8 (2)	H9A—C9—H9B	108.2
C5—N1—Mn1	119.1 (2)	C7—C8—C14	109.3 (3)
C17—N3—C21	116.0 (3)	C7—C8—H8A	109.8
C17—N3—Mn1	118.8 (2)	C14—C8—H8A	109.8
C21—N3—Mn1	125.1 (2)	C7—C8—H8B	109.8
C22—N4—C23	125.0 (3)	C14—C8—H8B	109.8
C22—N4—H4B	117.5	H8A—C8—H8B	108.3
C23—N4—H4B	117.5	C31—C30—C29	109.9 (3)
C6—N2—C7	125.2 (3)	C31—C30—H30A	109.7
C6—N2—H2B	117.4	C29—C30—H30A	109.7
C7—N2—H2B	117.4	C31—C30—H30B	109.7
C14—C12—C16	109.4 (3)	C29—C30—H30B	109.7
C14—C12—H12A	109.8	H30A—C30—H30B	108.2
C16—C12—H12A	109.8	C27—C25—C23	109.9 (3)
C14—C12—H12B	109.8	C27—C25—H25A	109.7
C16—C12—H12B	109.8	C23—C25—H25A	109.7
H12A—C12—H12B	108.3	C27—C25—H25B	109.7
C28—C27—C32	109.6 (3)	C23—C25—H25B	109.7
C28—C27—C25	109.7 (3)	H25A—C25—H25B	108.2
C32—C27—C25	108.6 (3)	C30—C31—C32	110.9 (3)
C28—C27—H27A	109.6	C30—C31—C26	109.9 (3)
C32—C27—H27A	109.6	C32—C31—C26	108.2 (3)
C25—C27—H27A	109.6	C30—C31—H31A	109.3
C23—C26—C31	109.9 (3)	C32—C31—H31A	109.3
C23—C26—H26A	109.7	C26—C31—H31A	109.3
C31—C26—H26A	109.7	C11—C16—C12	109.9 (3)
C23—C26—H26B	109.7	C11—C16—C10	109.6 (3)
C31—C26—H26B	109.7	C12—C16—C10	109.2 (3)
H26A—C26—H26B	108.2	C11—C16—H16A	109.4
C29—C28—C27	109.7 (3)	C12—C16—H16A	109.4
C29—C28—H28A	109.7	C10—C16—H16A	109.4
C27—C28—H28A	109.7	N3—C17—C18	123.7 (3)
C29—C28—H28B	109.7	N3—C17—H17A	118.1
C27—C28—H28B	109.7	C18—C17—H17A	118.1
H28A—C28—H28B	108.2	C34—C35—C36	117.3 (3)
O1—C6—N2	123.6 (3)	C34—C35—C38	117.7 (3)
O1—C6—C3	120.1 (3)	C36—C35—C38	124.8 (3)
N2—C6—C3	116.3 (3)	C38—N6—C39	126.9 (3)
C4—C3—C2	117.4 (3)	C38—N6—H6A	116.5
C4—C3—C6	123.4 (3)	C39—N6—H6A	116.5
C2—C3—C6	119.1 (3)	C33—C34—C35	119.7 (4)
C28—C29—C30	110.1 (3)	C33—C34—H34A	120.2
C28—C29—C24	108.8 (3)	C35—C34—H34A	120.2
C30—C29—C24	109.2 (3)	N6—C39—C41	109.9 (3)
C28—C29—H29A	109.5	N6—C39—C42	106.9 (3)
C30—C29—H29A	109.5	C41—C39—C42	109.4 (3)
C24—C29—H29A	109.5	N6—C39—C40	111.9 (3)
C13—C15—C11	110.3 (3)	C41—C39—C40	109.9 (3)
C13—C15—C9	109.4 (3)	C42—C39—C40	108.8 (3)
C11—C15—C9	109.5 (3)	N5—C37—C36	124.4 (4)
C13—C15—H15A	109.2	N5—C37—H37A	117.8
C11—C15—H15A	109.2	C36—C37—H37A	117.8
C9—C15—H15A	109.2	O3—C38—N6	124.2 (3)
C18—C19—C20	116.8 (3)	O3—C38—C35	119.5 (3)
C18—C19—C22	125.3 (3)	N6—C38—C35	116.3 (3)
C20—C19—C22	117.9 (3)	C45—C44—C43	109.3 (3)
N4—C23—C26	111.3 (3)	C45—C44—H44A	109.8
N4—C23—C24	111.0 (3)	C43—C44—H44A	109.8
C26—C23—C24	110.2 (3)	C45—C44—H44B	109.8
N4—C23—C25	107.8 (3)	C43—C44—H44B	109.8
C26—C23—C25	108.0 (3)	H44A—C44—H44B	108.3
C24—C23—C25	108.4 (3)	C35—C36—C37	118.7 (4)
C16—C10—C7	109.7 (3)	C35—C36—H36A	120.7
C16—C10—H10A	109.7	C37—C36—H36A	120.7
C7—C10—H10A	109.7	C37—N5—C33	116.3 (4)
C16—C10—H10B	109.7	C44—C45—C40	109.9 (3)
C7—C10—H10B	109.7	C44—C45—C46	109.1 (3)
H10A—C10—H10B	108.2	C40—C45—C46	109.7 (3)
N2—C7—C10	112.4 (3)	C44—C45—H45A	109.3
N2—C7—C9	106.7 (3)	C40—C45—H45A	109.3
C10—C7—C9	108.8 (3)	C46—C45—H45A	109.3
N2—C7—C8	110.5 (3)	C43—C41—C39	109.5 (3)
C10—C7—C8	109.7 (3)	C43—C41—H41A	109.8
C9—C7—C8	108.6 (3)	C39—C41—H41A	109.8
N1—C5—C4	123.5 (3)	C43—C41—H41B	109.8
N1—C5—H5A	118.3	C39—C41—H41B	109.8
C4—C5—H5A	118.3	H41A—C41—H41B	108.2
C5—C4—C3	119.7 (3)	C48—C43—C41	109.7 (3)
C5—C4—H4A	120.2	C48—C43—C44	109.7 (3)
C3—C4—H4A	120.2	C41—C43—C44	109.2 (3)
N1—C1—C2	123.5 (3)	C48—C43—H43A	109.4
N1—C1—H1A	118.3	C41—C43—H43A	109.4
C2—C1—H1A	118.3	C44—C43—H43A	109.4
N3—C21—C20	123.7 (3)	C48—C47—C42	109.4 (3)
N3—C21—H21A	118.1	C48—C47—C46	109.4 (3)
C20—C21—H21A	118.1	C42—C47—C46	110.1 (3)
C14—C13—C15	109.0 (3)	C48—C47—H47A	109.3
C14—C13—H13A	109.9	C42—C47—H47A	109.3
C15—C13—H13A	109.9	C46—C47—H47A	109.3
C14—C13—H13B	109.9	C47—C42—C39	109.7 (3)
C15—C13—H13B	109.9	C47—C42—H42A	109.7
H13A—C13—H13B	108.3	C39—C42—H42A	109.7
C31—C32—C27	109.1 (3)	C47—C42—H42B	109.7
C31—C32—H32A	109.9	C39—C42—H42B	109.7
C27—C32—H32A	109.9	H42A—C42—H42B	108.2
C31—C32—H32B	109.9	C47—C46—C45	108.9 (3)
C27—C32—H32B	109.9	C47—C46—H46A	109.9
H32A—C32—H32B	108.3	C45—C46—H46A	109.9
C16—C11—C15	109.4 (3)	C47—C46—H46B	109.9
C16—C11—H11A	109.8	C45—C46—H46B	109.9
C15—C11—H11A	109.8	H46A—C46—H46B	108.3
C16—C11—H11B	109.8	C45—C40—C39	109.1 (3)
C15—C11—H11B	109.8	C45—C40—H40A	109.9
H11A—C11—H11B	108.2	C39—C40—H40A	109.9
C13—C14—C12	110.4 (4)	C45—C40—H40B	109.9
C13—C14—C8	109.8 (3)	C39—C40—H40B	109.9
C12—C14—C8	109.0 (3)	H40A—C40—H40B	108.3
C13—C14—H14A	109.2	N5—C33—C34	123.6 (4)
C12—C14—H14A	109.2	N5—C33—H33A	118.2
C8—C14—H14A	109.2	C34—C33—H33A	118.2
C1—C2—C3	119.3 (3)	C43—C48—C47	109.5 (3)
C1—C2—H2A	120.4	C43—C48—H48A	109.8
C3—C2—H2A	120.4	C47—C48—H48A	109.8
C19—C18—C17	120.2 (3)	C43—C48—H48B	109.8
C19—C18—H18A	119.9	C47—C48—H48B	109.8
C17—C18—H18A	119.9	H48A—C48—H48B	108.2
C21—C20—C19	119.5 (3)		

Symmetry code: (i) −*x*, −*y*+2, −*z*.

### Hydrogen-bond geometry (Å, º)

**Table:**

*D*—H···*A*	*D*—H	H···*A*	*D*···*A*	*D*—H···*A*
N4—H4*B*···O1^ii^	0.86	2.18	3.024 (4)	168
N2—H2*B*···O3^iii^	0.86	2.25	3.064 (4)	157

Symmetry codes: (ii) *x*−1, *y*, *z*; (iii) *x*, *y*+1, *z*.
